# Oxidized DJ-1 Levels in Urine Samples as a Putative Biomarker for Parkinson's Disease

**DOI:** 10.1155/2018/1241757

**Published:** 2018-05-14

**Authors:** Jihoon Jang, Soyeon Jeong, Sung Ik Lee, Wongi Seol, Hyemyung Seo, Ilhong Son, Dong Hwan Ho

**Affiliations:** ^1^Department of Molecular and Life Sciences, Hanyang University, Ansan-si, Gyeonggido, Republic of Korea; ^2^InAm Neuroscience Research Center, Sanbon Medical Center, College of Medicine, Wonkwang University, Sanbon-ro, Gunpo-si, Gyeonggido, Republic of Korea; ^3^Department of Neurology, Sanbon Medical Center, College of Medicine, Wonkwang University, Sanbon-ro, Gunpo-si, Gyeonggido, Republic of Korea

## Abstract

Parkinson's disease (PD) is the second most common neurodegenerative disease. Oxidative stress is the most critical risk factor for neurodegenerative diseases, including Alzheimer's disease (AD) and Huntington's disease (HD). Numerous reports have demonstrated that oxidative stress aggravates cytotoxicity in dopaminergic neurons and accelerates the formation of protein inclusions. In addition, oxidative stress, such as 4-hydroxynonenal (HNE), oxidized protein, and dopamine quinone, are related to PD progression. *DJ-1* is a PD-causative gene, and it plays a pivotal role as a sensor and eliminator of oxidative stress. Several studies have shown that oxidized DJ-1 (OxiDJ-1) formation is induced by oxidative stress. Hence, previous studies suggest that oxidized DJ-1 could be a biomarker for PD. We previously reported higher DJ-1 levels in Korean male PD patient urine exosomes than male non-PD controls. We speculate that OxiDJ-1 levels in PD patient urine might be higher than that in non-PD controls. In this study, we established an ELISA for OxiDJ-1 using recombinant DJ-1 treated with H_2_O_2_. Using Western blot assay and ELISA, we confirmed an increase of OxiDJ-1 from HEK293T cells treated with H_2_O_2_. Using our ELISA, we observed significantly higher, 2-fold, OxiDJ-1 levels in the urine of Korean PD patients than in non-PD controls.

## 1. Introduction

Parkinson's disease is the second most common neurodegenerative disease, and its primary symptoms include tremor, bradykinesia, and rigidity. The progression of PD is associated with an increase in reactive oxygen species (ROS), including superoxide anion, hydrogen peroxide, hydroxyl radicals, singlet oxygen, and lipid peroxyl radicals [1]. Previous studies have revealed that 4-hydroxynonenal (HNE), a by-product of lipid peroxidation, is one of the most bioactive aldehydes, and oxidative stress initiates its production [2]. It has been implicated that oxidative modification of proteins is accelerated by increase of ROS [3, 4]. The synthesis of dopamine quinone (DAQ) is also mediated by the oxidation of dopamine, thereby initiating mitochondrial damage and generation of DAQ-conjugated cytosolic adducts [5, 6]. These features are closely related to PD pathology.

*DJ-1* is known as a PD-causative gene, and its function is associated with oxidative stress in PD [7]. A number of reports have demonstrated that DJ-1 senses ROS in the cytosol and conjugates ROS oxygen groups on its cysteine-106 residue [8, 9]. Loss of ROS scavenger function in DJ-1 showed enhanced cytotoxicity and increase of *α*-synuclein (*α*-syn) aggregates, which are known as a culprit of PD-pathogenesis [10–12]. Interestingly, a previous report proposed that oxidized DJ-1 (OxiDJ-1) would be an efficient biomarker for PD diagnosis [13]. Levels of DJ-1 in biofluids, such as cerebrospinal fluid (CSF), whole blood, plasma, serum, saliva, and urine, were investigated as a biomarker for PD (Table 1). There were higher levels of OxiDJ-1 in the erythrocytes of PD patients than in those of healthy subjects or medicated PD patients [13]. Intriguingly, MPTP, a drug that induces PD symptoms, increased OxiDJ-1 levels in mouse erythrocytes [14]. These evidences suggest that screening for oxidized DJ-1 levels in urine could be a convenient and efficient tool for diagnosing PD. As such, we established an enzyme-linked immunosorbent assay (ELISA) for examining levels of OxiDJ-1 in patient urine. Using this ELISA, we found significantly higher levels of OxiDJ-1 in PD patient urine compared to controls.

## 2. Materials and Methods

### 2.1. Cell Culture and Treatment

Human embryonic kidney 293T (HEK293T) cells were cultured in Dulbecco's medium (DMEM) high glucose (Cellgro, Herndon, VA, USA) supplemented with 10% fetal bovine serum (Cellgro) and 1% penicillin-streptomycin (GIBCO/BRL, Gaithersburg, MD, USA) for 24 h under 5% CO_2_ conditions. We then treated the cells with 100 *μ*M of H_2_O_2_ (Sigma-Aldrich, St. Louis, MO, USA) for 0, 1, and 3 h. The culture media were collected, and cells were lysed directly in 1X sample buffer.

### 2.2. Patient Urine Sampling

Our study was approved by the Institutional Review Board of Sanbon Medical Center, Wonkwang University (IRB2013-24). Urine samples were collected from 33 PD patients and 22 non-PD controls (Table 2) at the Department of Neurology, Sanbon Medical Center. The PD assessments, the unified Parkinson's disease rating (UPDR), and Hoehn and Yahr (HY) scales, were not used in this study. Instead, all PD patients were diagnosed by a certified neurologist, based on the UK Brain Bank criteria. Before starting our analysis, we excluded urine samples with proteinuria, hematuria, or a pH lower than 5.5. Two milliliters of patient urine samples were thawed slowly overnight at 4°C and then vortexed. Samples were treated with 1% Triton X-100 and 1X Protease Inhibitor Cocktail Set III (Gendepot, Barker, TX, USA) and centrifuged at 18,000× g for 30 min at 4°C. After centrifugation, the supernatant was concentrated by centrifugation of 10,000× g for 30 min at 4°C using a NANOSEP 3 kDa OMEGA filter (Pall Life sciences, Ann Arbor, MI, USA) prewashed with DBPS until the samples were concentrated 5-fold.

### 2.3. Western Blot Assay

For our Western blot assay, 40 *μ*l of concentrated patient urine was mixed with 10 *μ*l of 5X protein sample buffer and boiled at 95°C for 5 min. We followed the Western blot procedure as previously described [24]. All membranes were incubated with the following primary antibodies: anti-DJ-1 (1 : 1000, #2134, Cell signaling technology, Danvers, MA, USA), anti-Oxidized DJ-1 (1 : 500, ab169520, MJF-R16(66-5), Abcam, Cambridge, MA, USA), anti-TSG 101 (1 : 1000, ab83, Abcam), and anti-*β*-actin (1 : 1000, sc-47778, Santa Cruz, Dallas, TX, USA). The membranes were then incubated in goat anti-rabbit or -mouse IgG with horseradish peroxidase (1 : 5000, Jackson Immunoresearch, West grove, PA, USA. 111-035-003 or 115-035-003, resp.). Intensity of the bands was measured by the Multi Gauge V 3.0 program (Fuji photo Film, Tokyo, Japan).

### 2.4. Sandwich Enzyme-Linked Immunosorbent Assay (ELISA)

Recombinant His DJ-1 (His-DJ-1) was purchased from Sino biological (Beijing, China). To generate OxiDJ-1, His-DJ-1 was incubated with 5 mM H_2_O_2_ or equivalent vehicle (sterile distilled water (DW)) for 1.5 h at 37°C. The generated OxiDJ-1 was confirmed by Western blot assay and standards of His-DJ-1 with H_2_O_2_ which were freshly made just before ELISA. For ELISA to detect OxiDJ-1, we coated each well of a MaxiSorp flat-bottom 96 well plate (44-2404-21, Nunc, Roskilde, Denmark) with OxiDJ-1 antibody (0.5 *μ*g/ml) and 50 mM carbonate buffer overnight at 4°C. Each well was then washed 3 times with 200 *μ*l of 1X PBST and blocked with 150 *μ*l of SuperBlock T20 (Thermo Fisher Scientific, Waltham, MA, USA) for 1 h at RT on a shaker. After blocking, each well was washed 4 times with 200 *μ*l of PBST. Next, standard OxiDJ-1, patient and control urine samples were loaded into duplicate wells of the plate and incubated overnight at 4°C on a shaker. The wells were washed 4 times and then incubated in 100 *μ*l of DJ-1 antibody conjugated with HRP (0.7 *μ*g/ml, 60R-2218, Fitzgerald Industries International, Concord, MA, USA) in SuperBlock T20 for 1.5 h at RT on a shaker. After that, the plate was washed 5 times with 200 *μ*l of PBST. We then added 100 *μ*l of 3, 3′5, 5′-tetramethylbenzidine (Sigma-Aldrich) into each well and incubated them for 10 min in a dark container at RT on a shaker. Lastly, 50 *μ*l of 2 N HCl was added to each well, and the absorbance was measured by Synergy 2 (Biotek Instrument, Winooski, VT, USA) at a 450 nm wavelength.

### 2.5. Statistical Analysis

The graphs are presented as the mean ± SEM. The data were analyzed using Prism6 (GraphPad software, La Jolla, CA, USA). Each statistical analysis is described in detail in the figure legends.

## 3. Results

### 3.1. Establishment of an OxiDJ-1 Sandwich ELISA Using Recombinant DJ-1 Protein Treated with H_2_O_2_

**Figure 1:**
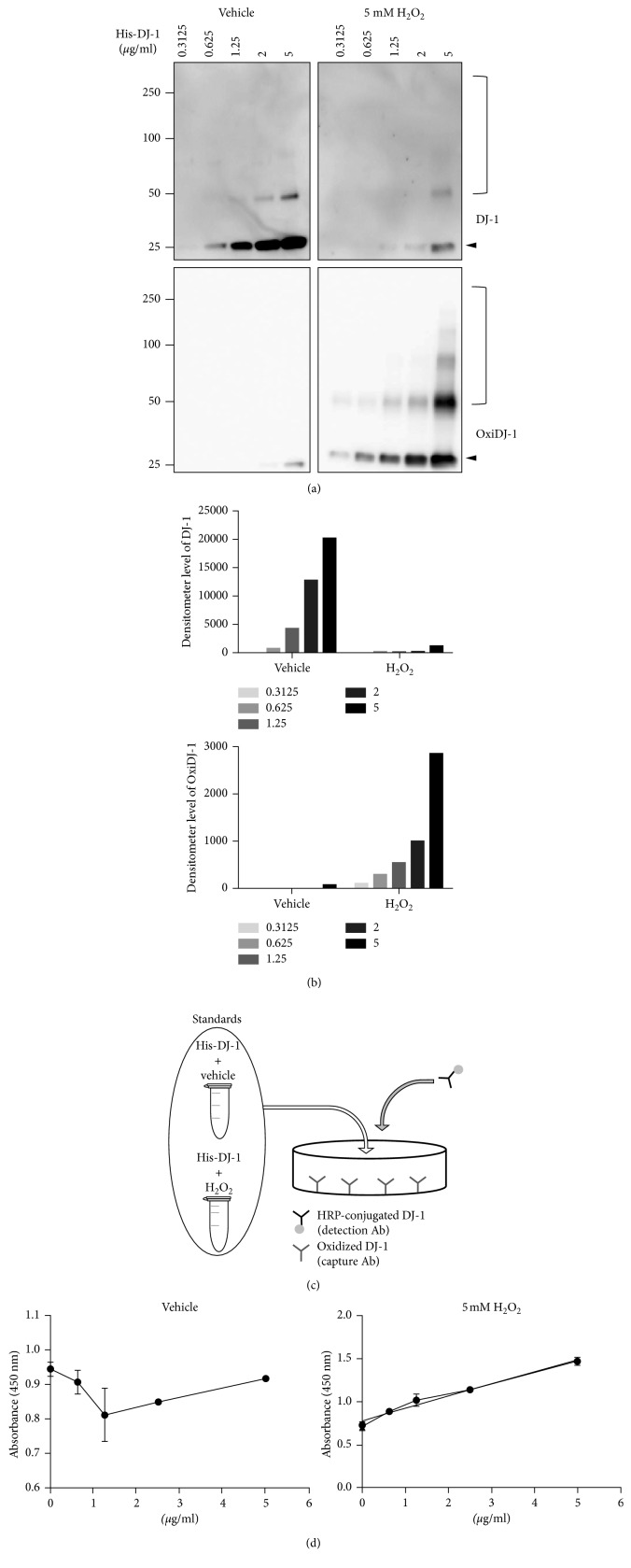
Establishment of the OxiDJ-1 ELISA. (a) Western blot analysis of OxiDJ-1. To generate OxiDJ-1, His-DJ-1 was incubated with 5 mM H_2_O_2_ or equivalent vehicle (sterile DW) for 1.5 h at 37°C and subjected to the Western blot analysis. (b) A quantitative analysis using a densitometer of the Western blot results in high molecular weight (HMW), or monomer was indicated by closed bracket or arrowhead, respectively. (c) A schematic diagram representing our OxiDJ-1 ELISA. (d) The standard curve obtained from the OxiDJ-1 ELISA. The ELISA was performed with an oxidized DJ-1 antibody as a capture antibody and HRP-conjugated DJ-1 antibody as a detection antibody using His-DJ-1 pretreated with H_2_O_2_ or DW as standard proteins.

In order to establish an OxiDJ-1 sandwich ELISA, we generated H_2_O_2_-mediated OxiDJ-1 by treatment of recombinant His-DJ-1 protein with 5 mM H_2_O_2_ for 1.5 h to use as a standard protein for ELISA. We confirmed that H_2_O_2_-treated His-DJ-1 showed a distinct increase in OxiDJ-1 and a gradual decrease in DJ-1 levels when DJ-1 concentration was gradually increased by Western blot analysis (Figures 1(a) and 1(b)). However, His-DJ-1 treated with DW, a vehicle control, had decreased or undetectable OxiDJ-1 levels (Figures 1(a) and 1(b)), while increased DJ-1 levels were detected. These data clearly confirm the activity of the OxiDJ-1 antibody. In addition, the formation of high-molecular weight DJ-1 aggregates seems to be facilitated by H_2_O_2_ treatment, as expected (Figure 1(a)).

We then designed a sandwich ELISA for OxiDJ-1, which was composed of oxidized DJ-1 and HRP-conjugated DJ-1 antibodies. Using the developed ELISA, we obtained an elaborate standard curve which had a gradual increase in H_2_O_2_-treated His-DJ-1 concentration, but not in DW-treated His-DJ-1 which had fluctuating absorbance (Figure 1(c)). These results suggest that our developed OxiDJ-1 sandwich ELISA could be a promising tool to measure OxiDJ-1 levels.

### 3.2. Validation of OxiDJ-1 Sandwich ELISA Using HEK293T Treated with H_2_O_2_

**Figure 2:**
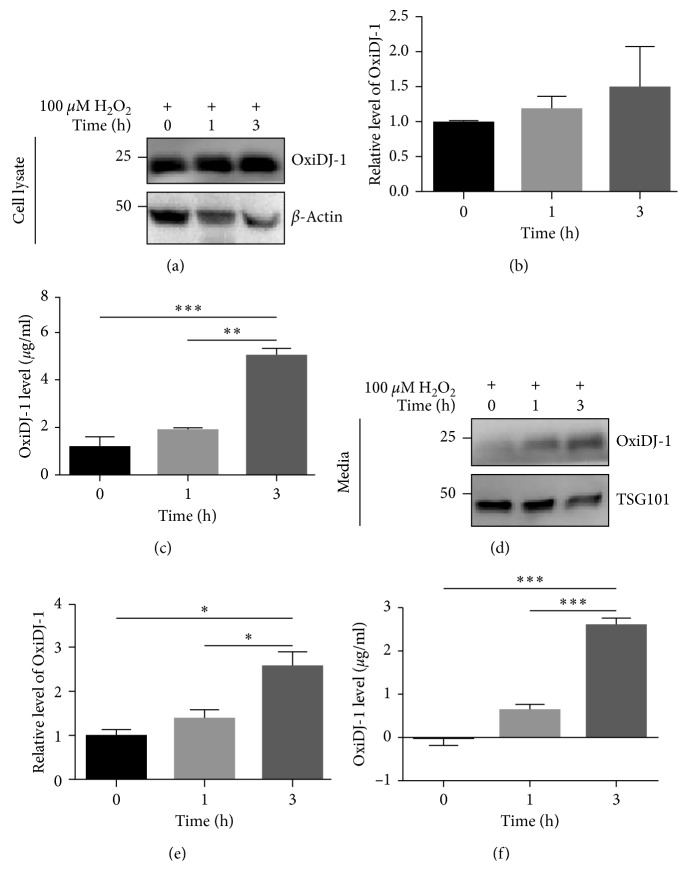
Detection of OxiDJ-1 treated with H_2_O_2_ in HEK293T cell lysates or cultured media. HEK293T cells were treated with 100 *μ*M H_2_O_2_ for 0, 1, and 3 h, and their cell lysates (a) or culture media (d) were subjected to Western blot assay. A quantitative analysis of cellular (b) or media (e) OxiDJ-1 levels, which were normalized by their *β*-actin (b) or TSG101 (e) levels, was done. Increased OxiDJ-1 levels in cell lysates (c) or media (f) were detected by ELISA. *n*=3, duplication for ELISA, One-way ANOVA, ^*∗*^ *p* < 0.05, ^*∗∗*^ *p* < 0.01, ^*∗∗∗*^ *p* < 0.001.

Previous studies have demonstrated that H_2_O_2_ treatment can induce OxiDJ-1 formation in the intracellular space and increase OxiDJ-1 levels in the extracellular space [26, 27]. To induce the formation of OxiDJ-1 in cells, HEK293T cells were treated with H_2_O_2_ for the indicated times and OxiDJ-1 levels of both the cell lysates and culture media were measured by both Western blot and ELISA. In the Western blot assay, increased OxiDJ-1 levels were observed in cell-lysates in a time-dependent manner, though it was not statistically significant (Figures 2(a) and 2(b)). The OxiDJ-1 ELISA of the lysates showed significantly higher OxiDJ-1 levels after the 3 h treatment than after only the 0 or 1 h treatment (Figure 2(c)). Moreover, both Western blot and ELISA of the culture media exhibited significantly higher OxiDJ-1 levels in the 3 h treatment than the 0 or 1 h treatment (Figures 2(d)–2(f)). These data support that our OxiDJ-1 ELISA is sensitive enough to detect live cell-derived OxiDJ-1. In addition, our results suggest that OxiDJ-1 is secreted in the culture medium.

### 3.3. Investigations of OxiDJ-1 Levels in Urine Samples of Korean PD Patients

**Figure 3:**
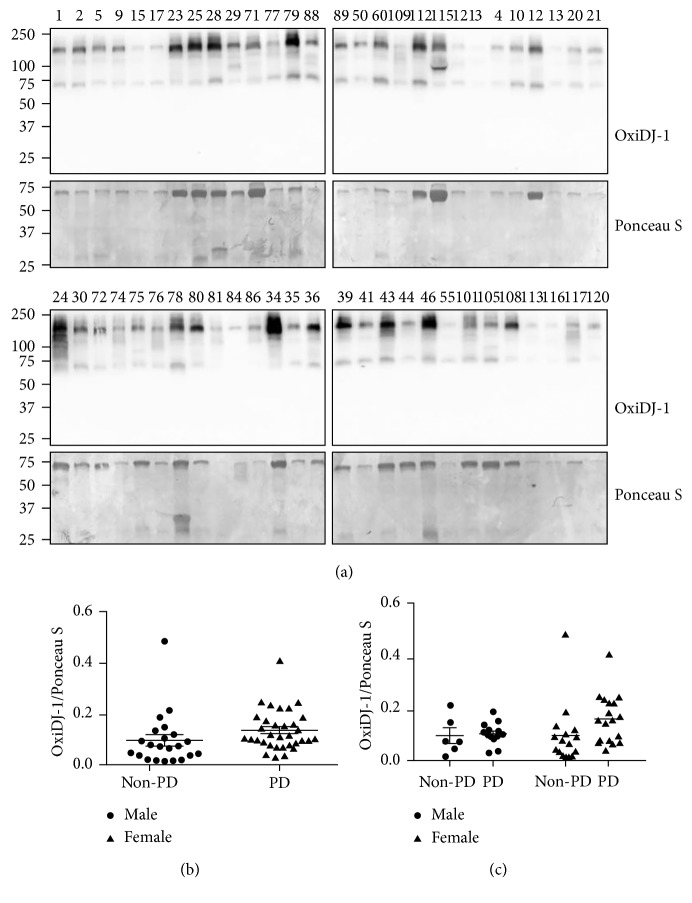
Comparison of OxiDJ-1 levels from patient urine samples using Western blot analysis. (a) Urine samples of non-PD controls (34, 35, 36, 39, 41, 43, 44, 46, 50, 55, 60, 101, 105, 108, 109, 112, 113, 115, 116, 117, 120, and 121) or PD patient (1, 2, 3, 4, 5, 9, 10, 12, 13, 15, 17, 20, 21, 23, 24, 25, 28, 29, 30, 71, 72, 74, 75, 76, 77, 78, 79, 80, 81, 84, 86, 88, and 89) were subjected to Western blot. The numbers at the top of the images are the patient numbers. (b) OxiDJ-1 levels were normalized with total protein levels determined by Ponceau S staining, and their ratio was diagramed. Student's *t*-test was used for this analysis. (c) Gender-grouped OxiDJ-1 levels were analyzed. All comparisons with one-way ANOVA were not significant.

**Figure 4:**
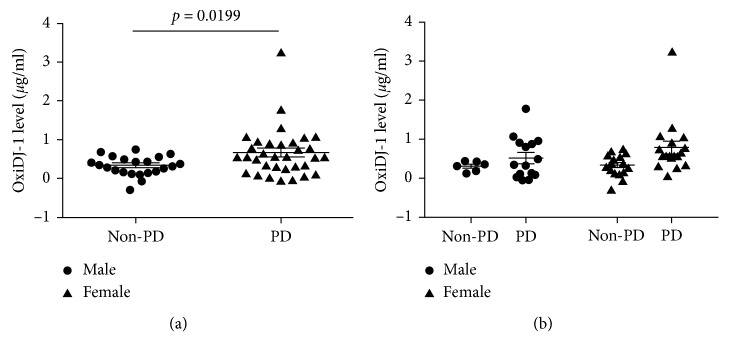
Detection of OxiDJ-1 from urine samples using ELISA. (a) OxiDJ-1 levels in urine were significantly increased in PD patients compared to non-PD controls. Student's *t*-test was used for this analysis, and *p* value is described in the graph. (b) OxiDJ-1 levels in urine were divided by gender. One-way ANOVA was used for statistical analysis.

Next, we tested the presence of OxiDJ-1 in human urine. A previous study used proteomic analysis to confirm that DJ-1 is secreted in human urine via exosomes [28]. Another study reported the presence of OxiDJ-1 in RBCs of human PD patients [13]. However, to date, there are no reports on the presence of OxiDJ-1 in human biofluids. In our study, Western blot assays of non-PD control or PD patient urine samples detected OxiDJ-1, but there was no significant difference in the amount of OxiDJ-1 between PD and non-PD participants (Figures 3(a) and 3(b)). Even after looking for gender-specific relevance within the two groups, we did not find any distinct differences (Figure 3(c)). Strikingly, when we used the established OxiDJ-1 ELISA, we found that PD patients had significantly 2-fold higher levels of OxiDJ-1 levels in their urine than non-PD controls (Figure 4(a)). Thus, our data confirms the presence of OxiDJ-1 in human urine.

There was also no significant gender-specific relevance within patient groups in OxiDJ-1 ELISA (Figure 4(b)). We also found no correlation between OxiDJ-1 level and PD onset age or duration (Supplementary Figure 1). We found some samples with negative quantities of OxiDJ-1 measuring at an absorbance lower than the interpolated standard of zero. This might be due to the formation of precipitates in their samples during ELISA, which might have reduced their antigen-antibody reaction or interrupted the OxiDJ-1 binding to the capture antibody. Taken together, our results support that investigating OxiDJ-1 levels in human urine samples could serve as a putative PD diagnostic tool. To clarify our findings, further studies with a larger number of samples are necessary.

## 4. Discussion

Oxidative stress is a highly evaluated risk factor of PD progression [1]. Increased ROS in dopaminergic neurons can modify cytosolic proteins via chemical conjugation [4]. HNE is produced from oxidation of lipids with polyunsaturated omega-6 acyl groups, such as arachidonic or linoleic acid, by lipid peroxidase [2, 29]. It has been implicated that HNE is conjugated with α-syn, thereby generating HNE-modified α-syn oligomers [30, 31]. Furthermore, PD patients showed higher levels of HNE-modified *α*-syn oligomers in the substantia nigra (SN) [32, 33]. Previous studies have reported that extensive oxidation of lipofuscin could aggravate proteasome activity and cause cytosolic aggregates [34, 35]. Inhibited proteasome activity is associated with *α*-synucleinopathy [36]. Other studies have demonstrated that DAQ can conjugate with *α*-syn to form unstructured adducts, and further, increased DAQ can exacerbate the survival of PC12, a rat dopaminergic neuronal cell line, via mitochondrial dysfunction [37, 38]. This evidence suggests that removal of oxidative stress via ROS-sensing molecules, such as chaperones, heat shock proteins, and DJ-1, is the most adequate therapeutic strategy for PD. In addition, cellular or extracellular OxiDJ-1 levels could be utilized as an indicator of oxidative stress levels.

As summarized in Table 1, numerous studies have investigated the use of DJ-1 as a PD biomarker. Several studies showed that DJ-1 levels in CSF [15, 18], plasma [16], and saliva [25] were increased in PD than the comparative groups. But other studies reported that DJ-1 levels were not different in serum samples from PD and control cases [17] and in CSF samples from parkinsonian syndrome and control groups [22]. On the other hand, contradictory levels of DJ-1 in CSF and saliva of PD were reported [20]. X. Lin et al. suggested that two types of HNE-modified DJ-1 isoform showed a distinguishable level in whole blood of PD [21]. Oxidative stress-mediated HNE might be associated with oxidation of DJ-1. OxiDJ-1 increased in unmedicated PD than medicated PD or healthy group [13]. Considering the molecular function of DJ-1 against oxidative stress, unmodified total DJ-1 might not be a suitable biomarker for PD, because the DJ-1 level showed inconsistent results depending on applications of biofluids or analytical methods. We assumed that oxidative stress-mediated modification of DJ-1 would be a hopeful approach for a diagnostic tool of PD.

Elimination of ROS by DJ-1 is accompanied by oxidation of DJ-1, itself [8]. A number of studies have shown that DJ-1 forms aggregates in the cytoplasm [39, 40]. We also observed increased OxiDJ-1 aggregates after treatment of His-DJ-1 with H_2_O_2_ (Figure 1(a)). Analyzing OxiDJ-1 levels in urine using Western blot assay also showed that most OxiDJ-1 in urine formed aggregates whereas monomeric OxiDJ-1 was barely detected after extreme long exposure (Figure 3(a)). We did not see any aggregated OxiDJ-1 or DJ-1 in cell lysates or media in the Western blot assay (data not shown). In HEK293T, OxiDJ-1 levels detected by the ELISA were consistent with those from the Western blot, but the differences were clearer in the ELISA (Figure 2). We only observed significant differences in OxiDJ-1 levels between PD and non-PD participants in the ELISA, not in the Western blot assay (Figures 3 and 4), suggesting that our ELISA is more sensitive than the Western blot assay.

Our previous study demonstrated that there were no significant differences in DJ-1 levels in urine exosomes during different times of day [24]. Further, only Korean male PD patients showed increased DJ-1 protein levels in their urine exosomes. However, DJ-1 proteins existed in both heated-soluble washing fractions, which are used for our analysis, and in retentate fractions, which were not analyzed in our previous study because of their low protein concentrations [24]. Therefore, in this study, we used the whole urine sample after incubating it with Triton X-100 detergent and concentrating it by filtration to acquire all proteins in the exosomes. This approach enabled us to detect the exact levels of total OxiDJ-1 in the urine sample of male and female patients. We were able to observe the presence of OxiDJ-1 in human urine and significant differences in OxiDJ-1 concentration between PD and non-PD participants. Although DJ-1 levels of Korean male PD patients were higher than those of female PD patients in our previous study, OxiDJ-1 levels exhibited no such difference (Supplementary Figure 2).

Consistent with previous reports, our study demonstrates that DJ-1 and OxiDJ-1 levels could be used as PD biomarkers in various materials from human. Using an OxiDJ-1 ELISA, we tried to observe differences in OxiDJ-1 levels in urine samples between PD patients and non-PD controls. However, it is important to note that in non-PD controls, OxiDJ-1 levels might be affected by other undiagnosed clinical conditions, including diabetes, hypertension, hyperlipidemia, and stroke. To verify the increase of OxiDJ-1 in PD patients, a future study with more PD patients and healthy, aged non-PD controls as well as healthy, young cohorts is necessary. Our finding might provide a key step towards finding an efficient diagnostic tool for PD.

## 5. Conclusion

We observed a 2-fold increase of OxiDJ-1 levels in the urine of Korean PD-patients compared to those in non-PD controls using our OxiDJ-1 ELISA. Thus, total OxiDJ-1 levels in human urine could be a feasible biomarker to diagnose PD.

## Figures and Tables

**Table 1:** Summary of clinical studied examining DJ-1 and OxiDJ-1 as possible biomarkers.

Material	Method	Outcome feature	Reference
CSF	WB^a^	DJ-1 levels were significantly higher in PD.	[15]
Plasma	WB	DJ-1 levels in PD were higher than those in controls.	[16]
Serum	ELISA^b^	DJ-1 shows similar levels in PD and in controls.	[17]
RBC	ELISA	OxiDJ-1 levels in unmedicated PD were higher than medicated PD or healthy control.	[13]
CSF	Luminex assay	DJ-1 levels were higher in PD than in control or AD.	[18]
Plasma	Luminex assay	DJ-1 was not a suitable biomarker of PD.	[19]
Saliva	Luminex assay	DJ-1 concentration was higher in PD.	[20]
CSF	Luminex assay	DJ-1 levels in PD were lower than in controls.	[20]
Whole blood	2D-PAGE, WB	DJ-1 levels were changed in the late stage of PD.	[21]
CSF	ELISA	DJ-1 levels did not change among Parkinsonian syndromes.	[22]
CSF	Luminex assay	There was no correlation between DJ-1 and striatal dopaminergic function.	[23]
Urine	WB	DJ-1 levels in PD males were significantly higher than those in controls.	[24]
Saliva	WB	DJ-1 was increased in PD and correlated with UPDRS score.	[25]
Urine	ELISA	OxiDJ-1 levels were higher in PD.	This study

^a^Western blot analysis; ^b^enzyme-linked immunosorbent assay.

**Table 2:** Summary of patient samples.

Gender	Non-PD		PD^a^	
	Male	Female	Male	Female
Number	6	16	15	18
Age (years)	71 ± 11.3	72 ± 9.6	76 ± 4.2	73 ± 7.0
Onset duration (years)	NA^b^	NA^b^	8.3 ± 2.87	7.3 ± 1.87

^a^PD patient information; PD diagnostic scores from the HY and UPDPS were not available; ^b^NA = not applicable.

## Supplementary Materials

Supplementary 1 Figure 1: correlation between onset duration of PD patients and OxiDJ-1 levels in patient urine. OxiDJ-1 levels were categorized by onset duration of PD. All onset duration groups were compared by one-way ANOVA and did not show any statistical differences.

Supplementary 2 Figure 2: comparison of OxiDJ-1 levels in urine by gender regardless of PD. OxiDJ-1 levels showed no statistical difference by Student's *t*-test between males and females.
