# Supplementary figures and images for: Oxidized DJ-1 Levels in Urine Samples as a Putative Biomarker for Parkinson's Disease
Source: Parkinsons Dis. 2018 May 14;2018:1241757. doi: 10.1155/2018/1241757 (PMC5985070; doi:10.1155/2018/1241757)

## Slide 1
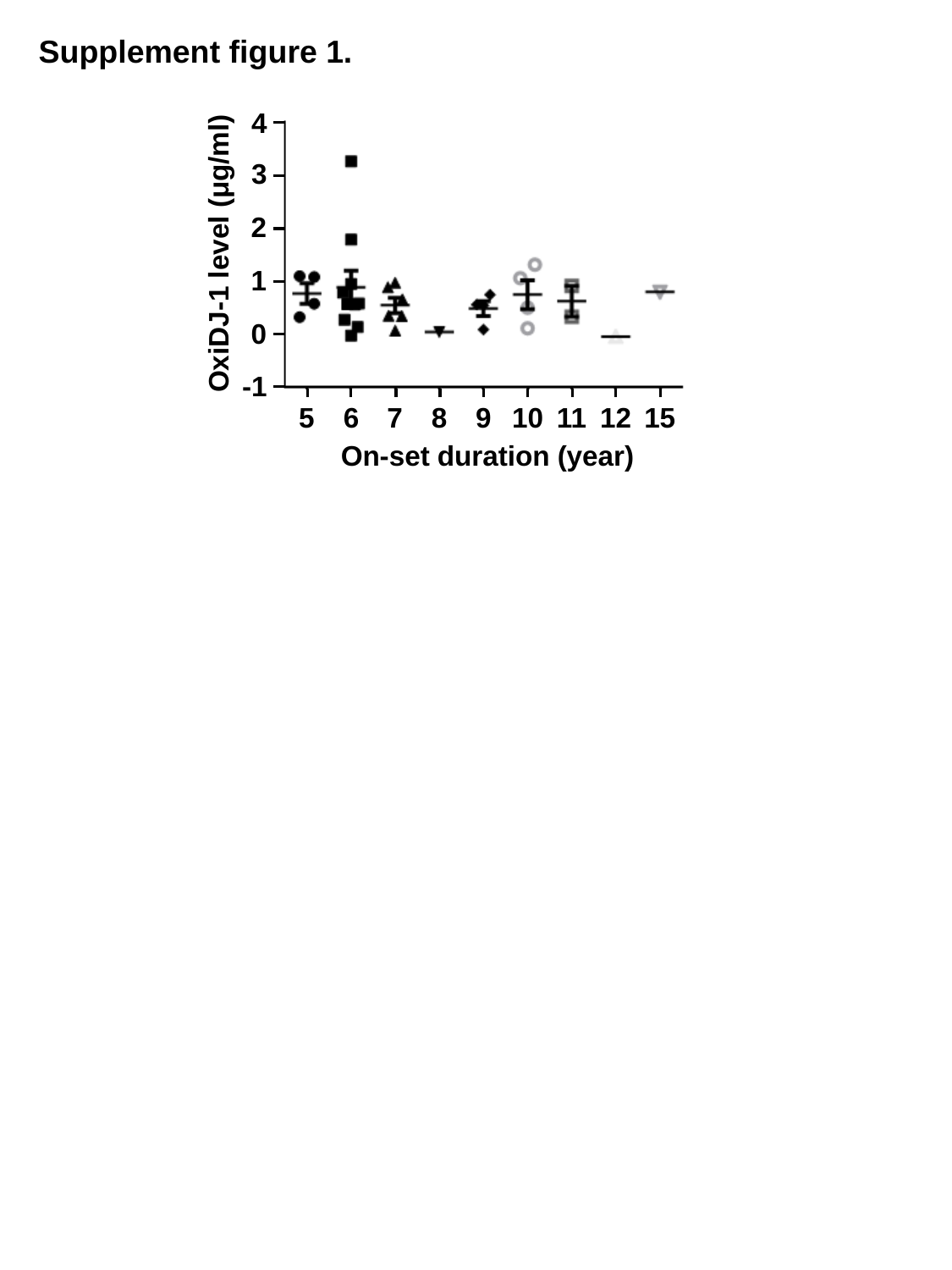

Supplement figure 1.
4
3
2
OxiDJ-1 level (μg/ml)
1
0
-1
5
6
7
8
9
10
11
12
15
On-set duration (year)

Supplement: Supplementary 1 — Figure 1: correlation between onset duration of PD patients and OxiDJ-1 levels in patient urine. OxiDJ-1 levels were categorized by onset duration of PD. All onset duration groups were compared by one-way ANOVA and did not show any statistical differences. [file 1241757.f1.pptx]

## Slide 1
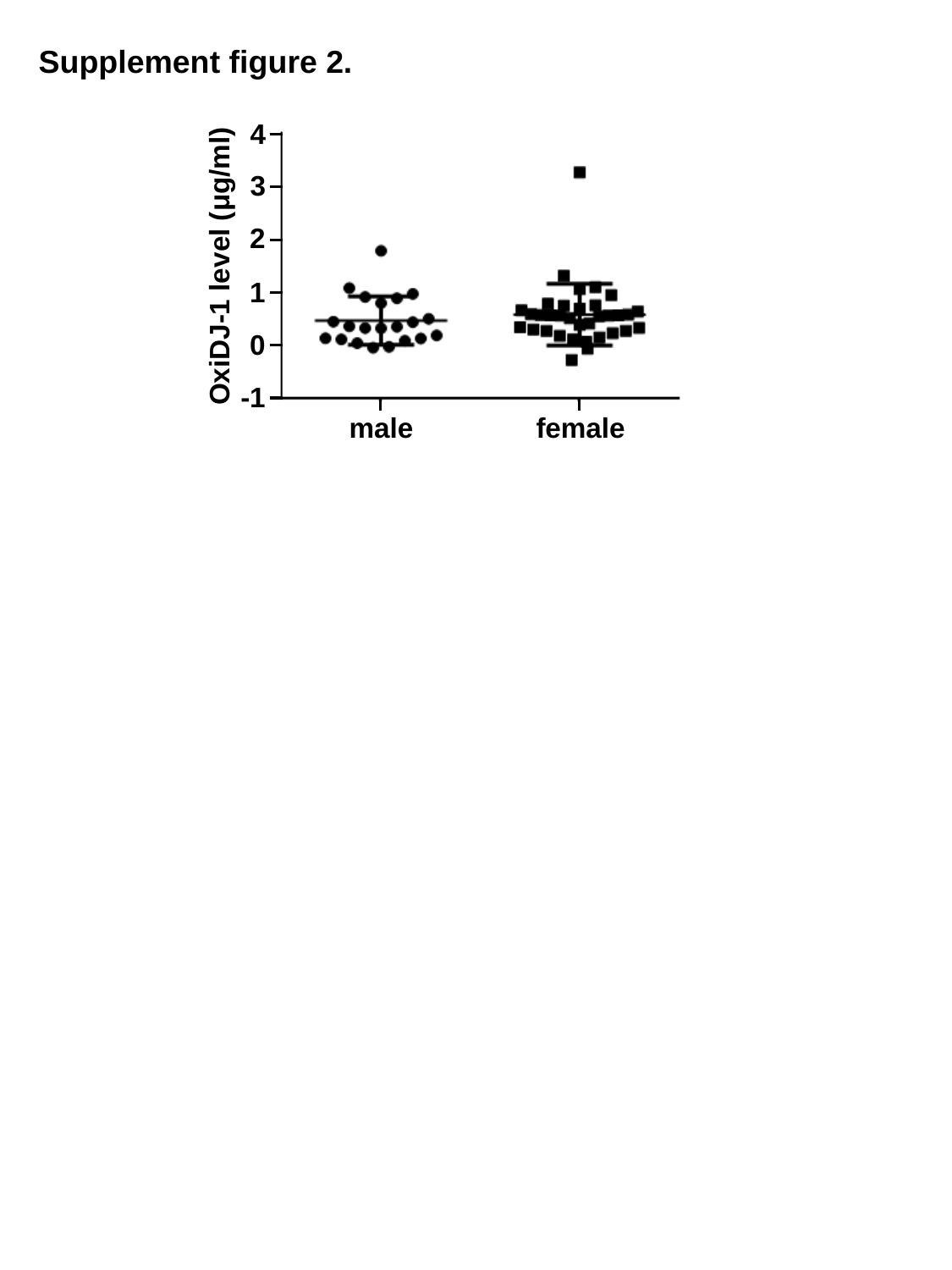

Supplement figure 2.
4
3
2
OxiDJ-1 level (μg/ml)
1
0
-1
male
female

Supplement: Supplementary 2 — Figure 2: comparison of OxiDJ-1 levels in urine by gender regardless of PD. OxiDJ-1 levels showed no statistical difference by Student's t-test between males and females. [file 1241757.f2.pptx]
